# Deciphering Pathways for Carotenogenesis in Haloarchaea

**DOI:** 10.3390/molecules25051197

**Published:** 2020-03-06

**Authors:** Micaela Giani, Jose María Miralles-Robledillo, Gloria Peiró, Carmen Pire, Rosa María Martínez-Espinosa

**Affiliations:** 1 Biochemistry and Molecular Biology Division, Agrochemistry and Biochemistry Department, Faculty of Sciences, University of Alicante, Ap. 99, 03690 Alicante, Spain; micaelagiani25@gmail.com (M.G.); jmmirallesrobledillo@gmail.com (J.M.M.-R.); carmen.pire@ua.es (C.P.); 2 Pathology Department and Research Unit; University General Hospital of Alicante; Pintor Baeza 12, 03010 Alicante, Spain; gloriapeiro@googlemail.com

**Keywords:** carotenogenesis, bacterioruberin, natural pigments, haloarchaea, carotenoids, antioxidant

## Abstract

Bacterioruberin and its derivatives have been described as the major carotenoids produced by haloarchaea (halophilic microbes belonging to the Archaea domain). Recently, different works have revealed that some haloarchaea synthetize other carotenoids at very low concentrations, like lycopene, lycopersene, cis- and trans-phytoene, cis- and trans-phytofluene, neo-β-carotene, and neo-α-carotene. However, there is still controversy about the nature of the pathways for carotenogenesis in haloarchaea. During the last decade, the number of haloarchaeal genomes fully sequenced and assembled has increased significantly. Although some of these genomes are not fully annotated, and many others are drafts, this information provides a new approach to exploring the capability of haloarchaea to produce carotenoids. This work conducts a deeply bioinformatic analysis to establish a hypothetical metabolic map connecting all the potential pathways involved in carotenogenesis in haloarchaea. Special interest has been focused on the synthesis of bacterioruberin in members of the *Haloferax* genus. The main finding is that in almost all the genus analyzed, a functioning alternative mevalonic acid (MVA) pathway provides isopentenyl pyrophosphate (IPP) in haloarchaea. Then, the main branch to synthesized carotenoids proceeds up to lycopene from which β-carotene or bacterioruberin (and its precursors: monoanhydrobacterioriberin, bisanhydrobacterioruberin, dihydrobisanhydrobacteriuberin, isopentenyldehydrorhodopsin, and dihydroisopenthenyldehydrorhodopsin) can be made.

## 1. Introduction

Carotenoids are widespread isoprenoid pigments in nature synthesized by bacteria, archaea, plants, algae, and yeasts [1,2]. Their main roles range from acting as accessory pigments in photosynthesis and as antioxidants affecting the regulation of gene expression [3]. Carotenoids are well-known for their color and potential beneficial effects on human health, and for that reason, they are frequently used in medical, nutraceutical, and pharmaceutical industries. These compounds are generally produced by chemical synthesis; however, the interest in the use of natural sources for carotenoid production is currently increasing [4,5,6]. 

Carotenoids are usually classified into three categories regarding the number of carbons present in their carotene backbones: C_30_, C_40_, and C_50_. Most carotenoids belong to the C_40_ group, as it is in the case of β-carotene. C_30_ and C_50_ carotenoids represent smaller percentages, especially the C_50_ category. C_50_ carotenoid structure results from the addition of two 5-carbon isoprene units to the C_40_ backbone [7]. So far, only the biosynthesis pathway of three C_50_ carotenoids has been determined: the ε-cyclic C_50_ carotenoid decaprenoxanthin [8], the β-cyclic carotenoid from *Corynebacterium poinsettiae* [9], and the γ-cyclic C_50_ carotenoid from *Micrococcus luteus* [10]. C_50_ carotenoids show higher antioxidative properties, given their longer conjugated double bonds and the presence of at least one hydroxyl group [11]. Therefore, this rare group of carotenoids is of interest to a wide range of industrial applications. The synthesis of C_50_ carotenoids has been barely studied, especially in the Archaea domain [7,12], whilst studies regarding plant, algae and bacteria C_30_ and C_40_ synthesis pathways are abundant in the literature [13,14,15]. 

Halophiles are a type of extremophile organism that requires high concentrations of salts for optimal growth, and that can be found in all three domains of life: Archaea, Bacteria, and Eukarya [16,17]. Within the Archaea domain, haloarchaea are those microbes showing mid or high requirements of salt to be alive. They are mainly grouped into two families: *Haloferacaceae* and *Halobacteriaceae* [18,19]. 

*Haloferax* is a halophilic genus that, in most cases, is pink-red colored given the production of C_50_ carotenoids, mainly bacterioruberin and its derivatives. These microorganisms also produce C_40_ carotenoids, although in lower percentages [7,16]. The acyclic C_50_ carotenoid bacterioruberin has been frequently observed in lipid membranes of halophilic archaea since these carotenoids are part of their mechanism of defense against salinity and temperature changes or sun radiation environments [20]. There is a clear consensus on the fact that most members of the families *Halobacteriaceae* and *Haloferacaceae* (to which *Haloferax* genus belong to) are able to synthesize C_50_ carotenoids, particularly bacterioruberin [20,21,22,23,24,25,26,27]. These characteristics make it interesting to suggest haloarchaeal species, such as *Haloferax* members, as good natural sources for the biosynthesis of carotenoids, particularly of those made of C_50_ backbones [16]. However, there is a lack of knowledge of carotenogenesis in haloarchaea. 

Falb and co-workers addressed a systematic metabolic reconstruction and comparative analysis of four completely sequenced genomes from haloarchaeal species in 2008 (*Halobacterium salinarum*, *Haloarcula marismortui*, *Haloquadratum walsbyi*, and the haloalkalophile *Natronomonas pharaonic*) [20]. In this study, the authors stated that the isoprenoid precursor isopentenyl pyrophosphate (IPP) is synthesized via the mevalonate pathway. It was also demonstrated that various isoprenoids detected in membranes of *H. salinarum* are synthesized by a series of condensation reactions with IPP, which is added in head-tail or head–head fashion, and through desaturase reactions. They also concluded that the enzymatic gene set for isoprenoid synthesis differs only slightly between the haloarchaea compared [20].

More recently, it was reported that lycopene is the branching point for bacterioruberin synthesis, although the reactions involved in this step in the *Haloferax* genus have not been brought to light yet. Moreover, experiments on *Haloarcula japonica* have led to the identification of three genes: *c0507*, *c0506*, and *c0505*; encoding respectively the following enzymes: carotenoid 3,4-desaturase (CrtD), a bifunctional lycopene elongase and 1,2-hydratase (LyeJ), and a C_50_ carotenoid 2″,3″-hydratase (CruF). These enzymes are responsible for the conversions from lycopene to bacterioruberin in *Haloarcula japonica* [12]. 

Considering these last findings and in order to optimize C_50_ carotenoid production using *Haloferax* sp. as a natural source, it is mandatory to assess the identification of the C_50_ biosynthesis pathway. The reason for using *Haloferax* sp. is because this is one of the best-characterized haloarchaeal genera from a biochemical and physiological point of view. Thus, this work conducts a deeply bioinformatic analysis to establish a hypothetical metabolic map connecting all the potential pathways involved in carotenogenesis in haloarchaea. A potential pathway for the synthesis of bacterioruberin in the genus *Haloferax* is also explored in detail. This proposal will open new research lines promoting the characterization of carotenogenesis in haloarchaea as well as the production of mutants able to overproduce carotenoids for biotechnological purposes like cosmetics, pharmacy and biomedicine.

## 2. Results

### 2.1. Reconstruction of a Metabolic Map for Global Carotenogenesis.

**Figure 1:**
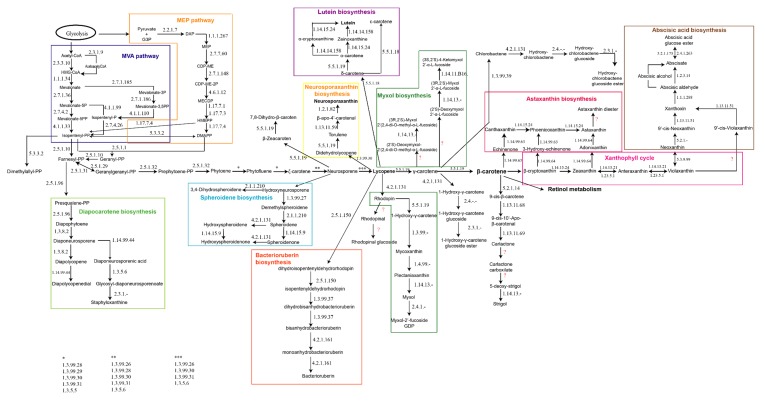
Metabolic map for global carotenogenesis. The enzymes catalyzing each reaction are indicated using their respective EC numbers (Enzyme commission numbers). Interrogation marks indicate unidentified enzymes.

Due to the lack of specific information related to carotenogenesis in haloarchaea, the first step in this work was to reconstruct a metabolic map including all the pathways described so far from different type of organisms (Eukarya, Bacteria, and Archaea), by integrating all the information available at BRENDA, KEGG, MetaCyc, NCBI, and Uniprot. The result of this integration is displayed in Figure 1.

The initiation of the carotenogenesis depends on the general isoprenoid biosynthetic pathway, along with a variety of other important natural substances such as steroids and gibberellic acid. Thus, the starting product required to synthetize all the isoprene derivatives is in general mevalonic acid, which is transformed into phosphorylated isoprene upon phosphorylation; this isoprene subsequently polymerizes. The cytosol-localized mevalonic acid (MVA) pathway delivers the basic isoprene unit isopentenyl diphosphate (IPP) in most of the organisms. However, in higher plants, this central metabolic intermediate is also synthesized by the plastid-localized methylerythritol phosphate (MEP) pathway. Both MVA and MEP pathways conspire through an exchange of intermediates and regulatory interactions (Figure 1, left panel) [28]. In the course of isoprene polymerization, the number and position of the double bonds are fixed [16]. Then, the conversion of two molecules of geranylgeranyl pyrophosphate (GGPP) to phytoene, a compound common to all C_40_ carotenogenic organisms, constitutes the first reaction unique to the carotenoid branch of isoprenoid metabolism [16,29]. From this step, slightly different reactions can be found in different organisms. As examples of this diversity, it is worthy of highlighting that organisms like anoxygenic photosynthetic bacteria, non-photosynthetic bacteria, and fungi desaturate phytoene either three or four times to yield neurosporene or lycopene, respectively. However, oxygenic photosynthetic organisms (cyanobacteria, algae, and higher plants) convert phytoene to lycopene via carotene in two distinct sets of reactions. At the level of neurosporene, lycopene, or β-carotene, the carotenoid biosynthesis pathways from different organisms branch to generate the significant diversity of carotenoids found in nature [16]. In photosynthetic organisms and tissues, the lipophilic carotenoid and bacteriochlorophyll (Bchl) or chlorophyll (Chl) pigment molecules associate non-covalently, but specifically, with integral membrane proteins. In non-photosynthetic organisms and tissues, carotenoids, often protein-bound, occur in cytoplasmic or cell wall membranes, oil droplets, crystals, and fibrils [16,30,31].

Thus, nine main groups of pathways related to carotenogenesis could be identified apart from other minor significant branches. Some of the main features of these nine groups are described following (Figure 1). At the level of farnesyl-PP, the diapocarotene biosynthesis pathway allows the synthesis of several compounds like diapophytoene, diapolycopene, or diaponeurosporene [32,33,34]. In this pathway, the carotenoid pigments are synthesized mainly via the desaturation of squalene rather than the direct synthesis of dehydrosqualene through the non-reductive condensation of prenyl diphosphate precursors, indicating the possible existence of a “squalene route” and a “lycopersene route” for C_30_ and C_40_ carotenoids, respectively [35]. In some bacteria, the following genes coding for the enzymes catalyzing this pathway have been described: 4,4’-diapophytoene synthase (*crtM*), 4,4’-diapophytoene desaturase (*crtNa*), 4,4’-diapolycopene ketolase (*crtNb*) and 4,4’-diapolycopene aldehyde oxidase (*crtNc*) [36].

From neurosporene (Figure 1), the main pathway can continue to lycopene, or two more different branches can be distinguished: one of them for the synthesis of zeacarotene and the other one the spheroidene biosynthesis pathway [37]. The compounds made through the spheroidene biosynthesis pathway are mainly related to antenna complexes of sulfur photosynthetic bacteria [38,39].

Lycopene is one of the key molecules in the global carotenogenesis because it is the precursor for several branches sustaining carotenogenesis. Thus, relevant carotenoids in nature like lutein and its precursors and derivatives [40,41,42], neurosporaxanthin, and the C_50_-carotenoid called bacterioruberin [43] are synthesized from lycopene. Neurosporaxanthin is a carboxylic xanthophyll synthesized from geranylgeranyl pyrophosphate through the activity of four enzymes, encoded by the genes *carRA, carB*, *carT,* and *carD* [44]. It is mainly produced by fungi, being *Fusarium* and *Neurospora,* the genera from which its synthesis has been characterized [45,46]. Bacterioruberin is the main carotenoid synthesized by microorganisms belonging to the haloarchaea group, and only a few species of *Micrococcus* genus apart from haloarchaea can produce it [43,47]. The following sections are focused on the elucidation of the potential pathways that may be involved in the synthesis of bacterioruberin in haloarchaea. Finally, lycopene is the precursor for the synthesis of the carotenoids of the rhodophinal series (Figure 1, central panels) [48].

Lycopene can also be converted to γ-carotene. This molecule acts as a precursor for the synthesis of myxol and its derivatives, γ-carotene glucosides, or β-carotene. Myxol biosynthesis has been less explored than other pathways related to carotenogenesis, and most of the results reported came from studies on cyanobacteria [49,50,51]. γ-carotene glucosides are poorly described in the literature, and they are usually described as “rare” carotenoids [34,52].

γ-carotene can be converted to β-carotene, which is also one of the key molecules in the global carotenogenesis because it is the precursor for the synthesis of astaxanthin, xanthophylls, and retinol. Retinol metabolism has not been included in Figure 1 due to its complexity and its connections to other pathways like those related to lipids, bile acids, and vitamins [53,54,55]. The xanthophyll cycle represents one of the important photoprotection mechanisms in plant cells [56] and it has been also related to structural stabilization of membranes and to modulation of protein-membrane function [57,58]. Astaxanthin is one of the carotenoids being successfully produced at large scale using *Haematococcus pluvialis* [59,60]. This carotenoid is a potent lipid-soluble keto-carotenoid with auspicious effects on human health. It protects organisms against a wide range of diseases with excellent safety and tolerability [61,62].

Finally, it is important to highlight that recently, a new carotenoid-derived pathway has been described as responsible for the synthesis of apocarotenoids, which include isoprenoids with important functions in plant-environment interactions such as the attraction of pollinators and the defense against pathogens and herbivores. Apocarotenoids also include volatile aromatic compounds that act as repellents, chemoattractants, growth stimulators, and inhibitors, as well as the phytohormones abscisic acid and strigolactones [63,64,65].

### 2.2. A Search of the Genes for Carotenogenesis in Haloarchaeal Genomes 

Considering all the information obtained for the reconstruction of the metabolic map for global carotenogenesis as well as the results already reported on the synthesis of carotenoids in haloarchaea, a two-step strategy was developed:

(i) Genes involved in the synthesis of bacterioruberin in *Haloarcula japonica* DSM 6131 were used as a query to look for their homologs in haloarchaeal available genomes at NCBI genome database [12]. Special interest has been paid on the following genomes because these are the haloarchaeal species better described from a biochemical point of view at the time of writing this work: *Haloferax mediterranei* ATCC3500, *Haloferax volcanii* DS2, *Haloferax gibbonsii* ARA6, *Haloarcula marismortui* ATCC43049, *Haloarcula hispanica* N601, *Halorubrum trapanicum* CBA1232, *Halorubrum ezzemoulense* Fb21, *Natronobacterium gregoryi* SP2, *Natronomonas moolapensis* 8.8.11 and *Haloquadratum walsbyi* C23.
(ii) All these sequences identified in the previous step were used as a query in BlastN to look for their homologs in other haloarchaeal genomes available at NCBI, in a one-to-one comparison. The enzymes encoded by these genes were identified using BlastX and Uniprot. Data about genes homology and identity are summarized in tables displayed in Supplementary Materials (for more details, see Section 4.2) (Supplementary materials: Tables S2–S14).

**Figure 2:**
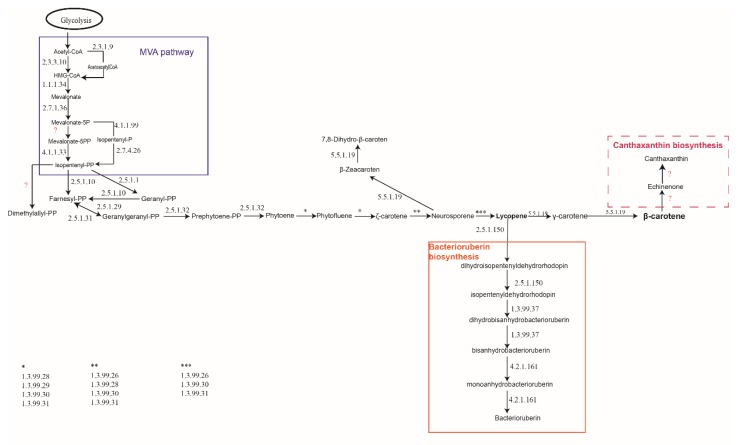
Carotenogenesis predicted in haloarchaea based on in silico analysis.

The results from this in silico analysis (Figure 2) demonstrated that in *H. mediterranei* genome, genes homologous to those involved in most of the carotenogenesis pathways were not identified (Figure 1). Haloarchaea synthesize terpenoid backbones through the mevalonate pathway, leading to the main branch of C_40_ carotenoids, common to the rest of organisms. At the lycopene level, the bacterioruberin synthesis pathway allows the synthesis of this C_50_ carotenoid and its derivatives. All of the studied haloarchaea share the genes involved in bacterioruberin synthesis, which were firstly described in *Haloarcula japonica* [12]. Thus, the pathway summarized in Figure 2 seems to be the main pathway sustaining the synthesis of these kinds of pigments in haloarchaea. Apart from bacterioruberin, haloarchaea could also be able to synthesize β-zeacaroten and 7,8-dihydro-β-carotene, using a minor branch from neurosporene (Figure 2, center).

Given this information, haloarchaea would synthesize almost exclusively bacterioruberin and its derivatives. However, few previous works have demonstrated that other carotenoids like canthaxanthin can be produced by *Haloferax alexandrinus* or *Halobacterium* species [66,67,68]. Nevertheless, the genes and enzymes involved in the synthesis of canthaxanthin were not clearly identified in neither of these species during this bioinformatic analysis. Another possible explanation would be the existence of undescribed pathways for the synthesis of other carotenoids, which would justify the allusion to them in some bibliography [68,69]. However, to this day, no other pigments have been clearly identified in haloarchaea.

### 2.3. Organization of the Genomes around the Genes Coding for the Enzymes Involved in the Synthesis of Bacterioruberin 

**Figure 3:**
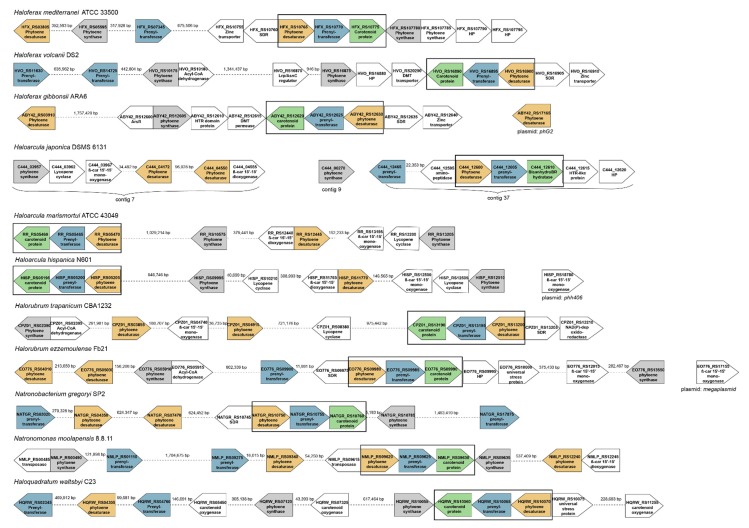
Organization of the genome around the genes involved in carotenogenesis in selected haloarchaeal species. Colors have been used to highlight those genes well conserved between species. Green color represents the gene coding for carotenoid-like protein; blue color represents the gene coding for prenyltransferase; grey color highlights the gene encoding phytoene synthase, and yellow color represents the gene coding phytoene desaturase. The main conserved gene cluster is framed in black.

The organization of ORFs around the genes coding for enzymes involved in the synthesis of bacterioruberin was examined in both, fully sequenced and drafts of haloarchaeal genomes, from eleven species that are representative of the genera *Haloarcula*, *Haloferax*, *Halorubrum*, *Haloquadratum*, *Natronobacterium,* and *Natronomonas.* Although there is little evidence of conservation in this region of the genome, all the genomes analyzed have at least one copy of genes coding the following enzymes: phytoene synthase, phytoene desaturase, prenyltransferase and a hypothetical protein annotated as “carotenoid biosynthesis protein” (Figure 3). All studied haloarchaea conserve a three-gene cluster containing one copy of genes coding phytoene desaturase, prenyltransferase and the already mentioned ‘’carotenoid biosynthesis protein’’. Apart from that, most species analyzed present also one or more other genes coding phytoene desaturase, phytoene synthase and prenyltransferase. However, these genes are usually nothing similar to the ones in the cluster nor among them. The total number of genes encoding the same enzyme is also variable, as, for example, in the case of the genus *Haloferax*, in which *Haloferax mediterranei* shows two genes for each of these enzymes, while *Haloferax volcanii* shows three genes coding prenyltransferase and only one for phytoene desaturase. On the opposite, *Haloferax gibbonsii* has three genes coding phytoene desaturase and only one for phytoene synthase and prenyltransferase. Regarding the genus *Haloarcula,* they all have a very conserved number of their genes, with the exception of *Haloarcula japonica*, which has two genes coding a prenyltransferase. Regarding the genus *Halorubrum,* most of the species analyzed present three copies of genes coding phytoene desaturase and two for phytoene synthase. However, *Halorubrum trapanicum* has one copy of the prenyltransferase gene, while *Halorubrum ezzemoulense* has two. *Natronobacterium gregoryi* and *Natronomonas moolapensis* have three genes coding for prenyltransferase and three encoding phytoene desaturases, but they differ in the number of phytoene synthase genes since *Natronobacterium* has one while *Natronomonas* has two *Haloquadratum walsbyi* presents two genes coding each of the three enzymes. 

Some other genes coding enzymes related to oxidative processes in carotenogenesis (β,β-carotene-15′-15′-monooxygenase and β-carotene-15′-15′-dioxygenase) have been observed in the genera *Haloarcula*, *Halorubrum,* and *Haloquadratum*.

Given the fact that most species present have more than one gene coding the same enzyme, a comparative analysis of the homology of the genes included in the clusters and those copies located in other areas of the genome has been carried out. The data obtained are summarized in Tables S2–S14 included in the Supplementary Material. Since *Haloferax mediterranei* is our model organisms, the results of this comparison in *Hfx. mediterranei* are discussed into detail hereunder:

(1) The analysis of the *Hfx. mediterranei* genome has revealed that this strain presents two genes encoding a phytoene desaturase: one inside the three-gene cluster and another one in another locus of the genome, both showing no resemblance between them. The one included in the three-gene cluster shows an identity between 68% and 87% with the correspondent gene of all the other species included in this study. On the other hand, when observing the gene located outside the cluster, it shares the highest homology (identity = 85.71%) with a phytoene desaturase-coding gene of *Haloferax gibbonsii*, also located outside the cluster; and shows also some identity with the correspondent gene of *Natronobacterium gregoryi* (67.13%) and with one of the two genes outside the cluster coding for this enzyme in *Natronomonas moolapensis* (65.67%), being both species haloalkalophiles.
(2) *Hfx. mediterranei* genome also has two non-homolog genes coding prenyltransferase: one being part of the cluster and the other one positioned upstream in the genome. The one inside the cluster shows a homology between 69% and 82% with the correspondent gene of all the other studied species; while the one located out of the cluster is very similar to the out-of-cluster gene coding the same enzyme of *Haloferax volcanii* (84.22%) and also, to the one of *Natronobacterium gregoryi* (66.81%) and *Natronomonas moolapensis* (69.58%).
(3) There is only one copy of the gene coding the unidentified ‘’carotenoid biosynthesis protein’’ in all the genomes studied. The one from *Hfx. mediterranei* genome shows a homology between 64% and 86% with the correspondent gene in the rest of the studied species, being the most similar *Hfx. volcanii* and *Hfx. gibbonsii*. However, the most remarkable hit is that this gene shows a 66.58% of homology with the gene coding the enzyme identified as bisanhydrobacterioruberin hydratase, also called C_50_ carotenoid 2′’,3′’-hydratase, in *Haloarcula japonica* [12].
(4) Finally, as a significant feature of the *Haloferax* genus, it is worth mentioning the presence of a gene coding a zinc transporter next to the carotenogenesis three-gene cluster in all the species of this genus. The possible implication of this gene is still unknown.

**Figure 4:**
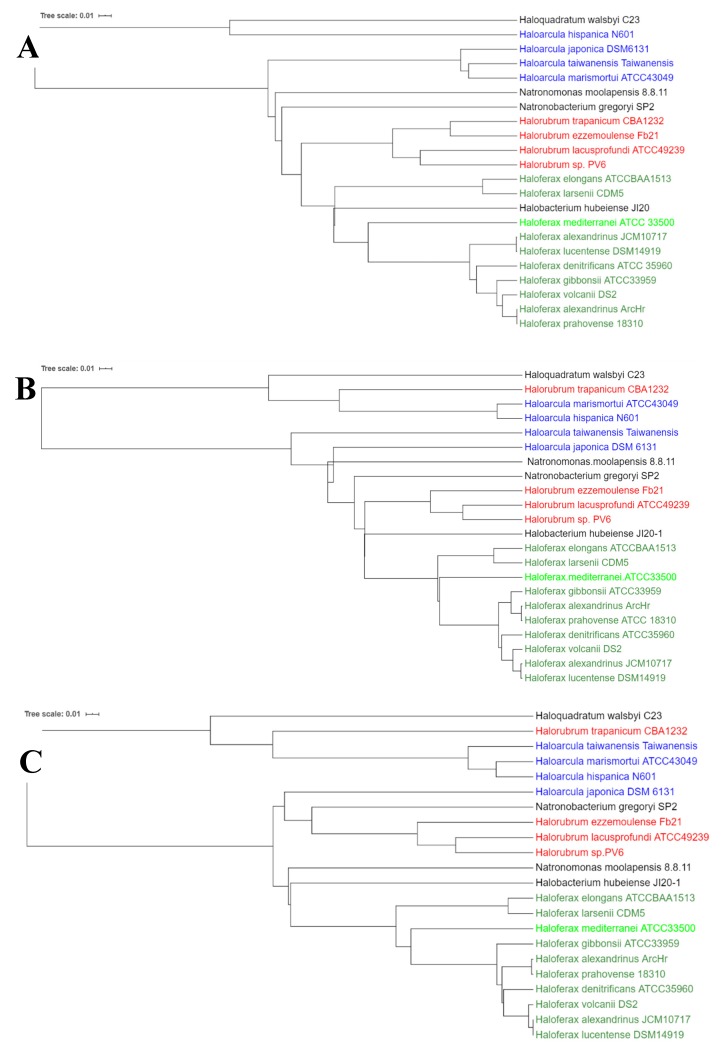
Unrooted phylogenetic tree of genes encoding the following enzymes: (**A**) Prenyltransferase; (**B**) phytoene desaturase; (**C**) putative bisanhydrobacterioruberin hydratase. Colors have been used to group species belonging to the same genus. Tree scale indicates the phylogenetic distance in branches. Trees were built with Clustal Omega (default settings) and annotation was done with iTol v4.

The considerable diversity in the organization of genes around those ORFs coding for carotenoid protein, prenyltransferase, phytoene desaturase, and phytoene synthase, and the existence of more than one copy for these genes in some of the analyzed species could be accounted for by the recent acquisition of those genes through horizontal gene transfer and subsequent recombination events, as it has also been reported for other genes like those related to denitrification in haloarchaea [70]. In order to get a complete view of the evolutionary relationship of carotenogenesis in selected haloarchaeal species, a phylogenetic tree for each of the three genes included in the cluster has been elaborated and analyzed (Figure 4). For all the three genes, it can be observed that in general, each of the sequences is highly conserved between species belonging to the same genus. The exception to this general pattern is the prenyltransferase-coding gene and the unidentified carotenoid protein-coding gene from *Halorubrum trapanicum*, which are closer to the genus *Haloarcula* than to its own. *Hfx. mediterranei* gene sequences are close to those from another member of the *Haloferax* genus; however, it is interesting to highlight that for the three genes *Hfx. mediterranei* constitute a branch. The pattern of branching in the phylogenetic trees in the case of *Hfx. mediterranei* could reflect a slightly different evolution of carotenogenesis or different frequencies of events of recombination and or/gene transfer.

## 3. Discussion

Since the first publication on carotenoids from archaea in the early sixties (last century) [71,72] more than 850 works have been published in indexed journals about this subject (PUBMED: data of access December the 10th, 2019; 856 publications identify using “carotenoids” and “archaea” as keywords). Most of these works addressed the characterization of the pigments synthesized by archaea, mainly haloarchaea, some others look for potential applications (due to their high antioxidant capability) and more recently, several works describe molecular engineering to optimize de production of some pigments in order to upscale their production [73,74,75,76]. However, there is still controversy about the nature of the pathways for carotenogenesis in haloarchaea, and consequently, the production of overpigmented mutants as cell factories for carotenoid production is still a limitation.

Considering that the number of haloarchaeal genomes fully sequenced has increased significantly during the last decade, and using plants, yeast, and cyanobacteria as model organisms from which carotenogenesis has been very well described so far, a three steps analysis has been conducted in order to elucidate key pathways involved in carotenogenesis in haloarchaea.

The first step was to reconstruct a metabolic map, including all the pathways described so far from a different types of organisms (involving Eukarya, Bacteria, and Archaea) (Figure 1). Secondly, the global map of carotenogenesis has been debugged through an in silico analysis to determine which pathways could be working in haloarchaea to sustain carotenogenesis. We analyzed around 100 genomes and, in particular, eleven species that are representative of the genera *Haloarcula*, *Haloferax*, *Halorubrum*, *Haloterrigena*, *Haloquadratum*, *Natronobacterium* and *Natronomonas* (Supplementary materials: Table S1). The results of the analysis suggest that although two components of the MVA pathway are often absent in archaeal genomes, the search for these missing elements led to the discovery of isopentenyl phosphate kinase (IPK) and phosphomevalonate decarboxylase (PMD). IPK and PMD carry out the two activities necessary to furnish the universal five-carbon isoprenoid building block, isopentenyl diphosphate (IPP) [77]. Thus, a functioning alternative MVA pathway could be the main pathway to provide isopentenyl-PP in haloarchaea from our results, which are in concordance with the results from other previous works [77,78,79].

Then, lycopene is obtained after a series of reactions in which geranylgeranyl-PP and prephytoene are produced. This main branch continues as a central metabolic of the backbone of carotenogenesis in which lycopene acts as a precursor (Figure 2). These results are in concordance with those described for *Haloarcula japonica* [12]. From lycopene, the major carotenoid produced by haloarchaea (bacterioruberin) is finally synthesized. Genes coding for the enzymes catalyzing reactions to produce β-carotene or β-zeacarotene have also been identified. So, on the base of nutritional status of the cells, and the physicochemical parameters fixed for cellular growth (temperature, radiation, pH, salt concentration, etc.), haloarchaea could synthetize phytoene, phytofluene, γ-carotene, β-carotene, neurosporene, lycopene, zeacarotene and bacterioruberin (and its derivatives and precursors) (Figure 2). The final concentration of each of those carotenoids, quantified from cellular extracts, will depend on the conditions of the culture as well as on the phase of growth in which the cells are harvested [80].

Some of the first studies on carotenoids in haloarchaea in the later seventies demonstrated that some haloarchaeal species could produce higher amounts of C_40_ carotenoids (lycopene and β-carotene) than C_50_ (bacterioruberin and its derivatives) when using glycerol as carbon source. Thus, the supplementation of the culture with up to 0.5% of glycerol stimulated carotenogenesis in *Halobacterium cutirubrum,* but the content of the major red pigment, bacterioruberin, was reduced fourfold and that of the minor red pigments, mono- and bisanhydrobacterioruberins, was also reduced but to a lesser extent [81]. Shortly after these discoveries, it was stated that the C_50_ bacterioruberin is made by the addition of a C_5_-isoprene unit to each end of the C_40_-lycopene chain, followed by the introduction of four hydroxyl groups [82]. 

In principle, all the genomes analyzed have at least one copy of genes coding the following enzymes: phytoene synthase, phytoene desaturase, prenyltransferase, and the hypothetical protein annotated as “carotenoid biosynthesis protein”. However, one of the main problems and limitations found during this study was the presence of ambiguous annotations regarding several genes. *Hfx. mediterranei* genome is an example of this limitation. Apparently, this species has two genes coding a phytoene desaturase, which are indeed nothing alike. One of these two genes, which is located outside the three-gene cluster, can be identified as a FAD-dependent oxidoreductase but is also annotated as an identical protein of phytoene desaturase. This conflict of annotation and the problems related to the incomplete or not completely accurate genome assembling have been previously described in other haloarchaeal studies [70]. Consequently, one of the main conclusions from this study is that more effort must be made in the next future to assembly haloarchaeal draft genomes and to review and update gene annotations in completely sequenced haloarchaeal genomes. 

Regarding the unidentified protein, a similarity of the sequence has been found to the gene coding the bisanhydrobacterioruberin hydratase or C_50_ carotenoid 2′’,3′’-hydratase enzyme of *Haloarcula japonica* [12]. However, much experimental research, including mutants, is needed to confirm the putative function of this gene in *Hfx. mediterranei.*

The presence of a gene coding a zinc transporter is a peculiar feature only found in species of the *Haloferax* genus. This transporter could be related to the fact that prenyltransferases are usually metalloenzymes that use zinc to catalyze their reactions [83,84]. It is still unknown if the prenyltransferases from haloarchaea present a zinc-site recognition moiety. Nevertheless, from the results of our in silico analysis using the ab initio ligand predictor IonCom, it can be assumed that the two prenyltransferases encoded in the genome of *Hfx. mediterranei* possess potential zinc recognition sites [85]. Another potential connection between this zinc transporter and carotenogenesis could be the “*bop*-gene regulon [69,86,87,88]. *bop* gene is part of a cluster of genes, which is tightly regulated by a sensor regulator called *bat* and potentially also by a small zinc-finger containing a protein called Brz [69]. *bop* gene codes for bacterio-opsin protein: part of the light-driven proton pump called bacteriorhodopsin [60,88]. However, this potential connection would not be related to *Hfx. mediterranei* because this species is not able to synthesize bacteriorhodopsin [89].

The phylogenetic relationship of the sequences of the three genes included in the cluster shows that each of the sequences is highly conserved between species belonging to the same genus, with the exception of those from *Halorubrum trapanicum*. In this case, sequences were closer phylogenetically to the genus *Haloarcula* than to its own in two out of three of the cluster genes. 

Finally, the presence of genes coding carotenoid oxygenases in *Haloarcula, Halorubrum,* and *Haloquadratum* could explain the more reddish pigmentation found in these genera when compared to other species such as *Haloferax mediterranei,* which show an intense pink color. 

In summary, although haloarchaea could synthesize other carotenoids apart from bacterioruberin (like β-carotene, lycopene, or in some species canthaxanthin), the major carotenoid is bacterioruberin and its relatives. The pathway deciphered in this work (Figure 2) sheds light on the knowledge of carotenogenesis in archaea and will contribute to the production of mutants over pigmented in order to upscale carotenoids production using archaea as biofactories [80,90]. Other issues like regulation of carotenogenesis [91] in haloarchaea and connections between carotenogenesis, synthesis of bacteriorhodopsin, and monooxygenases [69] should be explored into detail in the near future in order to understand the whole process making possible cellular pigmentation in this kind of microorganisms.

## 4. Materials and Methods 

### 4.1. Data Sample

To decipher pathways for carotenogenesis in haloarchaea, 100 haloarchaeal genomes (including fully sequenced genomes and drafts) available at NCB were analyzed (http://www.ncbi.nlm.nih.gov/genomes). Special interest was paid to the eleven following species because they are the best described from a physiological and biochemical point of view, up to the time of writing this work: *Haloferax mediterranei* ATCC3500, *Haloferax volcanii* DS2, *Haloferax gibbonsii* ARA6, *Haloarcula japonica* DSM6131, *Haloarcula marismortui* ATCC43049, *Haloarcula hispanica* N601, *Halorubrum trapanicum* CBA1232, *Halorubrum ezzemoulense* Fb21, *Natronobacterium gregoryi* SP2, *Natronomonas moolapensis* 8.8.11 and *Haloquadratum walsbyi* C23. 

### 4.2. In Silico Analysis of the Haloarchaeal Genomes.

A three steps strategy was designed to carry out an in silico analysis: i) reconstruction of a metabolic map including all the pathways described so far from different type of organisms (Eukarya, Bacteria, and Archaea), by integrating all the information available at BRENDA (https://www.brenda-enzymes.org/index.php), KEGG (https://www.genome.jp/kegg/), MetaCyc (https://metacyc.org/), Uniprot (https://www.uniprot.org/) and NCBI (https://www-ncbi-nlm-nih-gov/biosystems); ii) prediction of pathways involved in carotenogenesis in haloarchaeal: genes already described as sequences involved in the synthesis of bacterioruberin in haloarchaea were used as a query to look for their homologs in selected haloarchaeal genomes at NCBI [12], and the organization of the genome around those genes was analyzed deeply; iii) search of homologs to gene sequences coding for the enzymes represented in Figure 1 in order to identify the main pathways sustaining carotenogenesis in haloarchaea.

Given the observed variability of sequences between the conserved cluster of different species, the errors found in the gene annotation, and the difficulty to identify the correct ORF in some cases, we did not establish a threshold to find orthologs. For this reason, in order to identify the putative orthologs in all studied species, each gene was compared to each other to determine the percentage of identity between them, and therefore, the conserved cluster (S2-14). In general, in this one-to-one comparison, a minimum query cover of 80% and a minimum identity of 60% was considered to identify the gene as an ortholog. 

### 4.3. Bioinformatic Tools

BRENDA, KEGG, MetaCyc, and Uniprot databases were used to set up the metabolic map of carotenogenesis. NCBI Gene database was consulted to look for (i) genes previously related to bacterioruberin production; (ii) genes that encode the enzymes of the bacterioruberin pathway map in haloarchaeal genomes.

Blastn and BlastX online tools from NCBI (https://blast.ncbi.nlm.nih.gov/Blast.cgi) were used respectively to find homologous genes and their proteins in haloarchaeal genomes [92]. The default setting and organism filter were selected. Gene location and genetic context studies were carried out by means of the bioinformatic tool NCBI Sequence Viewer 3.33.0 (https://www.ncbi.nlm.nih.gov/tools/sviewer/).

Alignments of the sequences and phylogenetic tree have been done using Clustal Omega (https://www.ebi.ac.uk/Tools/msa/clustalo/) tool (web form) based on the HH algorithm described by Söding [93,94]. Default parameters were used. Annotation of the phylogenetic tree has been done with the online tool iTol v4 (https://itol.embl.de/) [95].

Zinc binding sites of *Haloferax mediterranei* prenyltransferases were analyzed using the IonCom server (https://zhanglab.ccmb.med.umich.edu/IonCom/) [85].

## Supplementary Materials

The following are available online at https://www.mdpi.com/1420-3049/25/5/1197/s1. A total of 14 tables are available as Supplementary Materials.
