# Supplementary material for: Deciphering Pathways for Carotenogenesis in Haloarchaea
Source: Molecules. 2020 Mar 6;25(5):1197. doi: 10.3390/molecules25051197 (PMC7179442; doi:10.3390/molecules25051197)
Supplement: Supplementary file 1 [file molecules-25-01197-s001.pdf]

SUPPLEMENTARY MATERIAL:

Table S1: Species included in the *in silico* analysis. Genome sequences were retrieved from the NCBI genome database (https://www.ncbi.nlm.nih.gov/genome/).

Genus	Species
Haloferax	*Haloferax mediterranei* ATCC 33500
	Haloferax mucosum PA12, ATCC BAA 1512
	Haloferax denitrificans ATCC 35960
	Haloferax prahovense DSM 18310
	Haloferax gibbonsii ARA6
	Haloferax gibbonsii ATCC 33959
	Haloferax lucentense DSM 14919
	Haloferax volcanii DS2
	Haloferax alexandrinus Arc Hr
	Haloferax alexandrinus JCM 10717
	Haloferax sulfurifontis M6, ATCC BAA 897
	Haloferax elongans ATCC BAA 1513
	Haloferax larsenii JCM 13917
	Haloferax larsenii CDM 5
	Haloferax sp. ATCC BAA 644
	Haloferax sp. ATCC BAA 645
	Haloferax sp. ATCC BAA 646
Halorubrum	*Halorubrum lipolyticum* DSM 21995
	Halorubrum trapanicum CBA1232
	Halorubrum saccharovorum DSM 1137
	Halorubrum aidingense JCM 13560
	Halorubrum sp. PV6
	Halorubrum sodomense DSM 3755
	Halorubrum tebenquichense DSM 14210
	Halorubrum hochstenium ATCC 700873
	Halorubrum xinjiangense CGMCC 13527
	Halorubrum californiensis DSM 19288
	Halorubrum litoreum JCM 13561
	Halorubrum coriense DSM 10284
	Halorubrum ezzemoulense Fb21
	Halorubrum terre JCM 10247
	Halorubrum distributum JCM 10118
	Halorubrum halophilum B8
	Halorubrum kocurii JCM 14978
	Halorubrum arcis JCM 13916
	Halobrum aquaticum CGMCC 16377
	Halorubrum lacusprofundi ATCC 49239
Haloarcula	*Haloarcula japonica* DSM 6131
	Haloarcula hispanica CGMCC 1.2049
	Haloarcula hispanica N601
	Haloarcula amylolytica JCM 13557
	Haloarcula salaria H5 DGR
	Haloarcula vallismortis ATCC-29715

	Haloarcula marismortui ATCC 43049
	Haloarcula argentinensis DSM 12282
	Haloarcula sinaiiensis ATCC 33800
	Haloarcula californiae ATCC 33799
	Haloarcula sp. CBA1115
	Haloarcula sp. BJGN 2, ATCC 33799
	Haloarcula taiwanensis Taiwanensis
Halopiger	*Halopiger aswanensis* DSM 13151
	Halopiger sp. IIH3
	Halopiger xanaduensis SH 6
	Halopiger sp. IIH2
	Halopiger salifodinae KCY07 B2
Halococcus	*Halococcus hamelinensis* 100A6
	Halococcus morrhuae DSM 1307
	Halococcus thailandensis JCM 13552
	Halococcus sediminicola CBA1101
	Halococcus salifodinae DSM 8989
	Halococcus saccharolyticus DSM 5350
	Halococcus sp. 197ª
Halobacterium	*Halobacterium jilantaiense* CGMCC 1.5337
	Halobacterium salinarum R1
	Halobacterium sp. JI20 1
	Halobacterium sp. DL1
Haladaptatus	*Haladaptatus paucihalophilus* DX253
	Haladaptatus cibarius D43
Haloterrigena	*Haloterrigena salina* JCM 13891
	Haloterrigena jeotgali A29
	Haloterrigena thermotolerans DSM 11522
	Haloterrigena limicola JCM 13563
	Haloterrigena hispanica CDM 6
	Haloterrigena turkmenica DSM 5511
Halobiforma	*Halobiforma nitratireducens* JCM 10879
	Halobiforma lacisalsi AJ5, JCM 12983
Halovivax	*Halovivax asiaticus* JCM 14624
	Halovivax ruber XH 70, DSM 18193
Halosarcina	*Halosarcina pallida* JCM 14848
Halosimplex	*Halosimplex carlsbadense* 2.9.1.
Haloquadratum	*Haloquadratum walsbyi* C23
Natronococcus	*Natronococcus jeotgali* DSM 18795
	Natronococcus amylolyticus DSM 10524
	Natronococcus occultus SP4
Natronobacterium	*Natronobacterium gregoryi* SP2
	Natronobacterium texcoconense DSM 24767
Natrinema	*Natrinema altunense* AJ2
	Natrinema gari JCM 14663
	Natrinema versiforme JCM 10478
	Natrinema pallidum DSM 3751
	Natrinema pellirubrum 157, JCM 10476
	Natrinema salaciae DSM 25055
	Natrinema sp. J7 1

	Natrinema sp. J7 2
Natronorubrum	*Natronorubrum texcoconense* DSM 25211
	Natronorubrum tibetense DSM 13204
	Natronorubrum sediminis CGMCC 1.8981
	Natronorubrum bangense JCM 10635
	Natronorubrum sulfidifaciens JCM 14089
Natronomonas	*Natronomonas moolapensis 8.8.11*
Natrialba	*Natrialba aegyptia* DSM 13077
	Natrialba asiatica DSM 12278
	Natrialba magadii ATCC 43099
	Natrialba hulunbeirensis JCM 10989
	Natrialba chahannaoensis JCM 10990
	Natrialba taiwanensis DSM 12281

Table S2: Gene comparison analysis output for the phytoene desaturase located inside the cluster (first part)

**Phytoene desaturase cluster**	***Hfx. mediterranei* HFX_RS10765**		***Hbr. trapanicum* CPZ01_RS13200**		***Hrc. marismortui* RR_RS05470**		***Hfx. volcanii* HVO_RS16900**		***N. gregoryi* NATGR_RS10750**		***H. walsbyi* HQRW_RS10070**	
	Query cover (%)	Identity (%)	Query cover (%)	Identity (%)	Query cover (%)	Identity (%)	Query cover (%)	Identity (%)	Query cover (%)	Identity (%)	Query cover (%)	Identity (%)
Hfx. mediterranei HFX_RS10765	-	-	98	77.93	95	73.82	100	87.23	97	76.65	99	68.40
Hbr. trapanicum CPZ01_RS13200	-	-	-	-	95	74.47	98	80.70	98	77.71	95	65.42
Hrc. marismortui RR_RS05470	-	-	-	-	-	-	96	74.57	97	72.93	95	63.71
Hfx. volcanii HVO_RS16900	-	-	-	-	-	-	-	-	97	76.39	96	67.65
N. gregoryi NATGR_RS10750	-	-	-	-	-	-	-	-	-	-	96	67.01
H. walsbyi HQRW_RS10070	-	-	-	-	-	-	-	-	-	-	-	-
Hfx. gibbonsii ABY42_RS12630	-	-	-	-	-	-	-	-	-	-	-	-
N. moolapensis NMLP_RS09620	-	-	-	-	-	-	-	-	-	-	-	-
Hbr. ezzemoulense EO776_RS09980	-	-	-	-	-	-	-	-	-	-	-	-
Hrc. hispanica HISP_RS11770	-	-	-	-	-	-	-	-	-	-	-	-

Table S3: Gene comparison analysis output for the phytoene desaturase located inside the cluster (second part)

**Phytoene desaturase cluster**	**Hfx gibbonsii ABY42_RS12630**		**N. moolapensis NMLP_RS09620**		**Hbr. ezzemoulense EO776_RS09980**		**Hrc hispanica HISP_RS11770**		**Hrc. japonica C444_RS12600**	
	Query cover (%)	Identity (%)	Query cover (%)	Identity (%)	Query cover (%)	Identity (%)	Query cover (%)	Identity (%)	Query cover (%)	Identity (%)
Hfx. mediterranei HFX_RS10765	100	87.29	94	72.97	98	77.29	94	71.49	95	73.26
Hbr. Trapanicum CPZ01_RS13200	98	81.85	96	76.16	100	92.75	95	72.09	95	74.04
Hrc. Marismortui RR_RS05470	96	75.03	97	74.95	96	75.28	97	71.46	100	93.15
Hfx. volcanii HVO_RS16900	100	95.81	95	75.05	98	80.46	95	73.29	95	74.14
N. gregoryi NATGR_RS10750	98	76.50	95	72.94	97	77.72	95	69.87	97	73.10
H. walsbyi HQRW_RS10070	96	67.51	93	64.93	94	66.11	57	67.07	92	64.03
Hfx. gibbonsii ABY42_RS12630	-	-	97	74.40	98	80.87	96	72.86	95	74.20
N. moolapensis NMLP_RS09620	-	-	-	-	97	75.52	97	71.05	97	75.07
Hbr. ezzemoulense EO776_RS09980	-	-	-	-	-	-	94	72.80	95	74.62
Hrc. hispanica HISP_RS11770	-	-	-	-	-	-	-	-	94	70.71

Table S4: Gene comparison analysis output for the phytoene desaturase located outside the cluster (first part).

**Phytoene desaturase (out of cluster)**	***Hfx. mediterranei* HFX_RS03805**		***Hrc. marismortui* RR_RS05470**		***Hbr. trapanicum* CPZ01_RS04915**		***Hbr. trapanicum CPZ01_RS03855***		***N. gregoryi* NATGR_RS04350**		***H. walsbyi* HQRW_RS04305**		***Hfx. gibbonsii ABY42_RS03910***		***Hfx. gibbonsii ABY42_RS17165***	
	Query cover (%)	Identity (%)	Query cover (%)	Identity (%)	Query cover (%)	Identity (%)	Query cover (%)	Identity (%)	Query cover (%)	Identity (%)	Query cover (%)	Identity (%)	Query cover (%)	Identity (%)	Query cover (%)	Identity (%)
Hfx. mediterranei HFX_RS03805	-	-	NS	NS	NS	NS	16	73.18	98	67.13	NS	NS	99	85.71	NS	NS
Hrc. marismortui RR_RS05470	-	-	-	-	96	66.55	85	74.42	10	79.41	37	65.51	NS	NS	NS	NS
Hbr. trapanicum CPZ01_RS04915	-	-	-	-	-	-	96	70.86	NS	NS	17	71.82	11	88.00	NS	NS
Hbr. trapanicum CPZ01_RS03855	-	-	-	-	-	-	-	-	NS	NS	52	72.57	21	75.96	17	70.79
N. gregoryi NATGR_RS04350	-	-	-	-	-	-	-	-	-	-	NS	NS	97	67.71	NS	NS
H. walsbyi HQRW_RS04305	-	-	-	-	-	-	-	-	-	-	-	-	NS	NS	NS	NS
Hfx. gibbonsii ABY42_RS03910	-	-	-	-	-	-	-	-	-	-	-	-	-	-	NS	NS
Hfx. gibbonsii ABY42_RS17165	-	-	-	-	-	-	-	-	-	-	-	-	-	-	-	-
N. moolapensis NMLP_RS09340	-	-	-	-	-	-	-	-	-	-	-	-	-	-	-	-
N. moolapensis NMLP_RS12240	-	-	-	-	-	-	-	-	-	-	-	-	-	-	-	-
Hbr. ezzemoulense EO776_RS04010	-	-	-	-	-	-	-	-	-	-	-	-	-	-	-	-
Hbr. ezzemoulense EO776_RS05030	-	-	-	-	-	-	-	-	-	-	-	-	-	-	-	-
Hrc. hispanica HISP_RS11770	-	-	-	-	-	-	-	-	-	-	-	-	-	-	-	-
Hrc. japonica C444_04172	-	-	-	-	-	-	-	-	-	-	-	-	-	-	-	-

Table S5: Gene comparison analysis output for the phytoene desaturase located outside the cluster (second part).

Phytoene desaturase(out of cluster)	***N. moolapensis NMLP_RS09340***		***N. moolapensis NMLP_RS12240***		***Hbr. ezzemoulense EO776_RS04010***		***Hbr. Ezzemoulense EO776_RS05030***		***Hrc. Hispanica HISP_RS11770***		***Hrc. japonica C444_04172***		***Hrc. japonica C444_RS04550***	
	Query cover (%)	*Identity (%)*	*Query cover (%)*	*Identity (%)*	*Query cover (%)*	*Identity (%)*	*Query cover (%)*	*Identity (%)*	*Query cover (%)*	*Identity (%)*	*Query cover (%)*	*Identity (%)*	*Query cover (%)*	*Identity (%)*
Hfx. mediterranei HFX_RS03805	*97*	*65.67*	*17*	*72.90*	*NS*	*NS*	*13*	*73.86*	*11*	*68.37*	*NS*	*NS*	*13*	*68.00*
Hrc. marismortui RR_RS05470	*NS*	*NS*	*97*	*70.20*	*95*	*66.53*	*97*	*68.50*	*97*	*71.46*	*NS*	*NS*	*97*	*70.97*
Hbr. trapanicum CPZ01_RS04915	*NS*	*NS*	*95*	*71.52*	*99*	*91.03*	*97*	*7076*	*94*	*68.69*	*13*	*94.44*	*93*	*67.47*
Hbr. trapanicum CPZ01_RS03855	*NS*	*NS*	*93*	*72.02*	*95*	*70.77*	*99*	*91.60*	*95*	*70.76*	*NS*	*NS*	*82*	*73.16*
N. gregoryi NATGR_RS04350	*95*	*65.54*	*NS*	*NS*	*12*	*71.43*	*11*	*84.62*	*NS*	*NS*	*NS*	*NS*	*15*	*68.03*
H. walsbyi HQRW_RS04305	*NS*	*NS*	*73*	*65.22*	*NS*	*NS*	*34*	*74.78*	*52*	*70.00*	*NS*	*NS*	*49*	*68.05*
Hfx. gibbonsii ABY42_RS03910	*96*	*67.76*	*17*	*75.70*	*10*	*83.33*	*14*	*86.21*	*19*	*68.57*	*NS*	*NS*	*14*	*73.85*
Hfx. gibbonsii ABY42_RS17165	*NS*	*NS*	*NS*	*NS*	*NS*	*NS*	*NS*	*NS*	*NS*	*NS*	*NS*	*NS*	*NS*	*NS*
N. moolapensis NMLP_RS09340	-	-	*17*	*79.17*	*16*	*74.58*	*NS*	*NS*	*16*	*86.96*	*NS*	*NS*	*11*	*100*
N. moolapensis NMLP_RS12240	-	-	-	-	*94*	*71.52*	*93*	*71.83*	*91*	*71.96*	*NS*	*NS*	*91*	*71.50*
Hbr. ezzemoulense EO776_RS04010	-	-	-	-	-	-	*96*	*70.76*	*94*	*68.69*	*NS*	*NS*	*93*	*67.72*
Hbr. ezzemoulense EO776_RS05030	-	-	-	-	-	-	-	-	*94*	*70.95*	*NS*	*NS*	*79*	*73.48*
Hrc. hispanica HISP_RS11770	-	-	-	-	-	-	-	-	-	-	*13*	*66.88*	*100*	*92.06*
Hrc. japonica C444_04172	-	-	-	-	-	-	-	-	-	-	-	-	*NS*	*NS*

Table S6: Gene comparison analysis output for the prenyltransferase located inside the cluster (first part).

**Prenyltransferase (cluster)**	***Hfx. mediterranei HFX_RS10770***		***Hrc. marismortui RR_RS05465***		***Hbr. trapanicum CPZ01_RS13195***		***Hfx. volcanii HVO_RS16895***		***N. gregoryi NATGR_RS10755***	
	Query cover (%)	Identity (%)	Query cover (%)	Identity (%)	Query cover (%)	Identity (%)	Query cover (%)	Identity (%)	Query cover (%)	Identity (%)
Hfx. mediterranei HFX_RS10770	-	-	89	70.90	88	72.85	95	82.19	92	69.17
Hrc. marismortui RR_RS05465	-	-	-	-	67	73.21	89	71.43	90	66.54
Hbr. trapanicum CPZ01_RS13195	-	-	-	-	-	-	83	75.09	86	70.46
Hfx. volcanii HVO_RS16895	-	-	-	-	-	-	-	-	89	72.44
N. gregoryi NATGR_RS10755	-	-	-	-	-	-	-	-	-	-
H. walsbyi HQRW_RS10065	-	-	-	-	-	-	-	-	-	-
Hfx. gibonsii ABY42_RS12620	-	-	-	-	-	-	-	-	-	-
N. moolapensis NMLP_RS09625	-	-	-	-	-	-	-	-	-	-
Hbr. ezzemoulense EO776_RS09985	-	-	-	-	-	-	-	-	-	-
Hrc. Hispanica HISP_RS05200	-	-	-	-	-	-	-	-	-	-

Table S7: Gene comparison analysis output for the prenyltransferase located inside the cluster (second part).

Prenyltransferase	***H. walsbyi HQRW_RS10065***		***Hfx gibbonsii ABY42_RS12625***		***N. moolapensis NMLP_RS09625***		***Hbr. ezzemoulense EO776_RS09985***		***Hrc. hispanica HISP_RS05200***		***Hrc. japonica C444_RS12605***	
	Query cover (%)	*Identity (%)*	*Query cover (%)*	*Identity (%)*	*Query cover (%)*	*Identity (%)*	*Query cover (%)*	*Identity (%)*	*Query cover (%)*	*Identity (%)*	*Query cover (%)*	*Identity (%)*
Hfx. mediterranei HFX_RS10770	*NS*	*NS*	*96*	*82.05*	*86*	*70.40*	*88*	*73.79*	*89*	*72.22*	*89*	*69.81*
Hrc. marismortui RR_RS05465	*44*	*71.55*	*88*	*71.86*	*93*	*71.82*	*69*	*71.70*	*100*	*94.92*	*100*	*92.2*
Hbr. trapanicum CPZ01_RS13195	*NS*	*NS*	*83*	*74.81*	*81*	*72.45*	*100*	*90.13*	*62*	*72.83*	*67*	*71.55*
Hfx. volcanii HVO_RS16895	*NS*	*NS*	*99*	*96.13*	*86*	*73.63*	*85*	*75.13*	*87*	*73.03*	*89*	*81.38*
N. gregoryi NATGR_RS10755	*NS*	*NS*	*92*	*72.06*	*90*	*70.64*	*92*	*70.29*	*88*	*67.39*	*86*	*66.58*
H. walsbyi HQRW_RS10065	-	-	*NS*	*NS*	*NS*	*NS*	*NS*	*NS*	*NS*	*NS*	*37*	*70.17*
Hfx. gibonsii ABY42_RS12620	-	-	-	-	*83*	*73.74*	*92*	*74.20*	*85*	*72.60*	*88*	*71.55*
N. moolapensis NMLP_RS09625	-	-	-	-	-	-	*87*	*72.25*	*92*	*73.13*	*94*	*71.09*
Hbr. ezzemoulense EO776_RS09985	-	-	-	-	-	-	-	-	*82*	*70.60*	*69*	*70.81*
Hrc. Hispanica HISP_RS05200	-	-	-	-	-	-	-	-	-	-	*100*	*90.96*

Table S8: Gene comparison analysis output for the prenyltransferase located outside the cluster (first part).

Prenyltransferase (out of cluster)	***Hfx. mediterranei HFX_RS07345***		***Hfx. volcanii HVO_RS14725***		***Hfx. volcanii HVO_RS11630***		***N. gregoryi NATGR_RS17875***		***N. gregoryi NATGR_RS03020***	
	Query cover (%)	*Identity (%)*	*Query cover (%)*	*Identity (%)*	*Query cover (%)*	*Identity (%)*	*Query cover (%)*	*Identity (%)*	*Query cover (%)*	*Identity (%)*
Hfx. mediterranei HFX_RS07345	-	-	*5*	*86.96*	*98*	*84.22*	*NS*	*NS*	*86*	*66.81*
Hfx.volcanii HVO_RS14725	-	-	-	-	*NS*	*NS*	*NS*	*NS*	*NS*	*NS*
Hfx.volcanii HVO_RS11630	-	-	-	-	-	-	*NS*	*NS*	*85*	*68.75*
N. gregoryi NATGR_RS17875	-	-	-	-	-	-	-	-	*NS*	*NS*
N. gregoryi NATGR_RS03020	-	-	-	-	-	-	-	-	-	-
H. walsbyi HQRW_RS04760	-	-	-	-	-	-	-	-	-	-
N. moolapensis NMLP_RS09275	-	-	-	-	-	-	-	-	-	-
N. moolapensis NMLP_RS01110	-	-	-	-	-	-	-	-	-	-
Hbr. ezzemoulense EO776_RS09900	-	-	-	-	-	-	-	-	-	-

Table S9: Gene comparison analysis output for the prenyltransferase located outside the cluster (second part).

Prenyltransferase (out of cluster)	***H. walsbyi HQRW_RS04760***		***N. moolapensis NMLP_RS09275***		***N. moolapensis NMLP_RS01110***		***Hbr. ezzemoulense EO776_RS09900***		***Hrc. japonica C444_RS12465***	
	Query cover (%)	*Identity (%)*	*Query cover (%)*	*Identity (%)*	*Query cover (%)*	*Identity (%)*	*Query cover (%)*	*Identity (%)*	*Query cover (%)*	*Identity (%)*
Hfx. mediterranei HFX_RS07345	*41*	*65.19*	*83*	*69.58*	*NS*	*NS*	*12*	*77.36*	*NS*	*NS*
Hfx.volcanii HVO_RS14725	*NS*	*NS*	*NS*	*NS*	*NS*	*NS*	*NS*	*NS*	*NS*	*NS*
Hfx.volcanii HVO_RS11630	*32*	*83.02*	*94*	*70.13*	*NS*	*NS*	*16*	*75*	*NS*	*NS*
N. gregoryi NATGR_RS17875	*NS*	*NS*	*NS*	*NS*	*NS*	*NS*	*NS*	*NS*	*NS*	*NS*
N. gregoryi NATGR_RS03020	*NS*	*NS*	*NS*	*NS*	*NS*	*NS*	*NS*	*NS*	*NS*	*NS*
H. walsbyi HQRW_RS04760	-	-	*NS*	*NS*	*NS*	*NS*	*NS*	*NS*	*NS*	*NS*
N. moolapensis NMLP_RS09275	-	-	-	-	*NS*	*NS*	*19*	*92*	*NS*	*NS*
N. moolapensis NMLP_RS01110	-	-	-	-	-	-	*10*	*90.91*	*NS*	*NS*
Hbr. ezzemoulense EO776_RS09900	-	-	-	-	-	-	-	-	*NS*	*NS*

Table S10: Gene comparison analysis output for the unidentified carotenoid biosynthesis protein located inside the cluster (first part).

Carotenoid biosynthesis protein	***Hfx. mediterranei HFX_RS10775***		***Hbr. trapanicum RS13190***		***Hrc. marismortui RR_RS05460***		***Haloferax volcanii HVO_RS16890***		***N. gregoryiNATGR_RS10760***	
	Query cover (%)	*Identity (%)*	*Query cover (%)*	*Identity (%)*	*Query cover (%)*	*Identity (%)*	*Query cover (%)*	*Identity (%)*	*Query cover (%)*	*Identity (%)*
Hfx. mediterranei HFX_RS10775	-	-	87	71.08	86	67.17	95	86.1	82	67.58
Hbr. trapanicum RS13190	-	-	-	-	78	71.51	86	74.72	82	72.38
Hrc. marismortui RR_RS05460	-	-	-	-	-	-	75	70.17	75	70.17
Haloferax volcanii HVO_RS16890	-	-	-	-			-	-	82	68.14
N. gregoryi NATGR_RS10760	-	-	-	-			-	-	-	-
H.walsbyi RS_10060	-	-	-	-			-	-	-	-
Hfx. gibbonsii ABY42_RS12620	-	-	-	-			-	-	-	-
N. moolapensis NMLP_RS09630	-	-	-	-			-	-	-	-
Halorubrum ezzemoulense EO776_RS09990	-	-	-	-			-	-	-	-
Hrc. hispanica HISP_RS05195	-	-	-	-			-	-	-	-

Table S11: Gene comparison analysis output for the unidentified carotenoid biosynthesis protein located inside the cluster (second part).

Carotenoid biosynthesis protein	***H.walsbyi RS_10060***		***Haloferax gibbonsii ABY42_RS12620***		***Natronomonas moolapensis NMLP_RS09630***		***Halorubrum ezzemoulense EO776_RS09990***		***Hrc. hispanica HISP_RS05195***		***Hrc. japonica C444_RS12610***	
	Query cover (%)	*Identity (%)*	*Query cover (%)*	*Identity (%)*	*Query cover (%)*	*Identity (%)*	*Query cover (%)*	*Identity (%)*	*Query cover (%)*	*Identity (%)*	*Query cover (%)*	*Identity (%)*
Hfx. mediterranei HFX_RS10775	87	64.29	92	85.92	87	67.55	90	71.66	87	66.16	86	66.58
Hbr. trapanicum RS13190	7%	71.83	86	73.92	83	68.32	100	100	76	72.43	79	70.96
Hrc. marismortui RR_RS05460	47	63.61	88	69.75	91	67.32	75	72.66	99	93.64	100	91.38
Haloferax volcanii HVO_RS16890	64	66.05	100	94.78	86	70.13	90	74.23	86	69.38	86	68.99
N. gregoryi NATGR_RS10760	16	68.92	82	68.87	82	67.78	83	72.66	75	72.01	82	70.92
H.walsbyi RS_10060	-	-	54	64.74	5	75.51	8	71.83	30	66.89	35	75
Hfx. gibbonsii ABY42_RS12620	-	-	-	-	88	69.55	90	73.92	88	68.69	86	68.74
N. moolapensis NMLP_RS09630	-	-	-	-	-	-	92	68.32	91	68.28	86	66.75
Halorubrum ezzemoulense EO776_RS09990	-	-	-	-	-	-	-	-	76	72.43	87	70.56
Hrc. hispanica HISP_RS05195	-	-	-	-	-	-	-	-	-	-	99	91.70

Table S12: Gene comparison analysis output for the phytoene synthase (first part).

Phytoene synthase	***Hfx. mediterranei* *HFX_RS10780***		***Hfx. mediterranei* *HFX_RS05595***		***Hbr. trapanicum* *CPZ01_RS02390***		***Hrc. marismortui* *RR_RS10575***		***Hrc. marismortui* *RR_RS13205***		***Hfx. volcanii* *HVO_RS16875***	
	Query cover (%)	*Identity (%)*	*Query cover (%)*	*Identity (%)*	*Query cover (%)*	*Identity (%)*	*Query cover (%)*	*Identity (%)*	*Query cover (%)*	*Identity (%)*	*Query cover (%)*	Identity (%)
Hfx. mediterranei HFX_RS10780	-	-	*NS*	*NS*	*NS*	*NS*	*NS*	*NS*	*73*	*68.22*	*100*	84.41
Hfx. mediterranei HFX_RS05595	-	-	-	-	*94*	*69.88*	*34*	*75.78*	*NS*	*NS*	*NS*	NS
Hbr. trapanicum CPZ01_RS02390	-	-	-	-	-	-	*81*	*65.06*	*NS*	*NS*	*15*	78.57
Hrc. marismortui RR_RS10575	-	-	-	-	-	-	-	-	*NS*	*NS*	*NS*	NS
Hrc. marismortui RR_RS13205	-	-	-	-	-	-	-	-	-	-	*78*	70
Hfx. volcanii HVO_RS16875	-	-	-	-	-	-	-	-	-	-	-	-
Hfx. volcanii HVO_RS10175	-	-	-	-	-	-	-	-	-	-	-	-
N. gregoryi NATGR_RS10785	-	-	-	-	-	-	-	-	-	-	-	-
H. walsbyi HQRW_RS10055	-	-	-	-	-	-	-	-	-	-	-	-
H.walsbyi HQRW_RS07120	-	-	-	-	-	-	-	-	-	-	-	-
Hfx. gibbonsii ABY42_RS12605	-	-	-	-	-	-	-	-	-	-	-	-
N. moolapensis NMLP_RS09635	-	-	-	-	-	-	-	-	-	-	-	-
N. moolapensis NMLP_RS00490	-	-	-	-	-	-	-	-	-	-	-	-
Hbr. ezzemoulense EO776_RS05910	-	-	-	-	-	-	-	-	-	-	-	-
Hbr. ezzemoulense EO776_RS13550	-	-	-	-	-	-	-	-	-	-	-	-
Hrc. hispanica HISP_RS09995	-	-	-	-	-	-	-	-	-	-	-	-
Hrc. hispanica HISP_RS12510	-	-	-	-	-	-	-	-	-	-	-	-
Hrc. japonica C444_RS06270	-	-	-	-	-	-	-	-	-	-	-	-

Table S13: Gene comparison analysis output for the phytoene synthase (second part).

**Phytoene synthase**	***Hfx. volcanii* *HVO_RS10175***		***N. gregoryi* *NATGR_RS10785***		***H. walsbyi* *HQRW_RS10055***		***H.walsbyi* *HQRW_RS07120***		***Hfx. gibbonsii* *ABY42_RS12605***		***N. moolapensis* *NMLP_RS09635***	
	Query cover (%)	*Identity (%)*	*Query cover (%)*	*Identity (%)*	*Query cover (%)*	*Identity (%)*	*Query cover (%)*	*Identity (%)*	*Query cover (%)*	*Identity (%)*	*Query cover (%)*	*Identity (%)*
Hfx. mediterranei HFX_RS10780	*NS*	*NS*	*71*	*68.99*	*69*	*67.09*	*NS*	*NS*	*100*	*84.22*	*68*	*70.24*
Hfx. mediterranei HFX_RS05595	*100*	*87.09*	*NS*	*NS*	*NS*	*NS*	*79*	*65.23*	*NS*	*NS*	*NS*	*NS*
Hbr. trapanicum CPZ01_RS02390	*95*	*73.20*	*NS*	*NS*	*NS*	*NS*	*NS*	*NS*	*NS*	*NS*	*NS*	*NS*
Hrc. marismortui RR_RS10575	*68*	*67.07*	*NS*	*NS*	*NS*	*NS*	*NS*	*NS*	*NS*	*NS*	*NS*	*NS*
Hrc. marismortui RR_RS13205	*NS*	*NS*	*77*	*68.54*	*NS*	*NS*	*NS*	*NS*	*78*	*70.10*	*79*	*67.71*
Hfx. volcanii HVO_RS16875	*NS*	*NS*	*77*	*70.62*	*69*	*68.07*	*NS*	*NS*	*100*	*93.63*	*79*	*70.62*
Hfx. volcanii HVO_RS10175	-	-	*NS*	*NS*	*NS*	*NS*	*79*	*64.81*	*NS*	*NS*	*NS*	*NS*
N. gregoryi NATGR_RS10785	-	-	-	-	*NS*	*NS*	*NS*	*NS*	*80*	*70.33*	*76*	*69.64*
H. walsbyi HQRW_RS10055	-	-	-	-	-	-	*NS*	*NS*	*49*	*71.57*	*NS*	*NS*
H.walsbyi HQRW_RS07120	-	-	-	-	-	-	-	-	*NS*	*NS*	*NS*	*NS*
Hfx. gibbonsii ABY42_RS12605	-	-	-	-	-	-	-	-	-	-	*78*	*70.34*
N. moolapensis NMLP_RS09635	-	-	-	-	-	-	-	-	-	-	-	-
N. moolapensis NMLP_RS00490	-	-	-	-	-	-	-	-	-	-	-	-
Hbr. ezzemoulense EO776_RS05910	-	-	-	-	-	-	-	-	-	-	-	-
Hbr. ezzemoulense EO776_RS13550	-	-	-	-	-	-	-	-	-	-	-	-
Hrc. hispanica HISP_RS09995	-	-	-	-	-	-	-	-	-	-	-	-
Hrc. hispanica HISP_RS12510	-	-	-	-	-	-	-	-	-	-	-	-
Hrc. japonica C444_RS06270	-	-	-	-	-	-	-	-	-	-	-	-

Table S14: Gene comparison analysis output for the phytoene synthase (third part).

Phytoene synthase	***N. moolapensis NMLP_RS00490***		***Hbr. ezzemoulense EO776_RS05910***		***Hbr. ezzemoulense EO776_RS13550***		***Hrc. hispanica HISP_RS09995***		***Hrc. hispanica HISP_RS12510***		***Hrc. japonica C444_RS06270***		***Hrc. japonica C444_03957***	
	Query cover (%)	*Identity (%)*	*Query cover (%)*	*Identity (%)*	*Query cover (%)*	*Identity (%)*	*Query cover (%)*	*Identity (%)*	*Query cover (%)*	*Identity (%)*	*Query cover (%)*	*Identity (%)*	*Query cover (%)*	*Identity (%)*
Hfx. mediterranei HFX_RS10780	*NS*	*NS*	*NS*	*NS*	*73*	*71.22*	*NS*	*NS*	*72*	*68.46*	*NS*	*NS*	*74*	*68.92*
Hfx. mediterranei HFX_RS05595	*95*	*70.16*	*94*	*70*	*NS*	*NS*	*60*	*67.12*	*NS*	*NS*	*67*	*67.12*	*NS*	*NS*
Hbr. trapanicum CPZ01_RS02390	*95*	*75.96*	*100*	*93.31*	*NS*	*NS*	*82*	*66.97*	*NS*	*NS*	*81*	*66.13*	*NS*	*NS*
Hrc. marismortui RR_RS10575	*77*	*66.15*	*70*	*65.85*	*NS*	*NS*	*100*	*94.03*	*NS*	*NS*	*100*	*92.71*	*NS*	*NS*
Hrc. marismortui RR_RS13205	*NS*	*NS*	*NS*	*NS*	*86*	*69.64*	*NS*	*NS*	*100*	*93.21*	*NS*	*NS*	*100*	*93.83*
Hfx. volcanii HVO_RS16875	*NS*	*NS*	*14*	*85.19*	*79*	*74.59*	*NS*	*NS*	*74*	*71.62*	*NS*	*NS*	*76*	*70.97*
Hfx. volcanii HVO_RS10175	*85*	*74.41*	*94*	*72.44*	*NS*	*NS*	*69*	*69.10*	*NS*	*NS*	*63*	*68.75*	*NS*	*NS*
N. gregoryi NATGR_RS10785	*NS*	*NS*	*NS*	*NS*	*87*	*71.91*	*NS*	*NS*	*79*	*67.88*	*NS*	*NS*	*75*	*68.78*
H. walsbyi HQRW_RS10055	*NS*	*NS*	*NS*	*NS*	*51*	*66.73*	*NS*	*NS*	*NS*	*NS*	*NS*	*NS*	*12*	*74.6*
H.walsbyi HQRW_RS07120	*61*	*64.64*	*NS*	*NS*	*NS*	*NS*	*17*	*73.73*	*NS*	*NS*	*NS*	*NS*	*NS*	*NS*
Hfx. gibbonsii ABY42_RS12605	*NS*	*NS*	*NS*	*NS*	*82*	*74.81*	*NS*	*NS*	*77*	*71.60*	*NS*	*NS*	*75*	*71.59*
N. moolapensis NMLP_RS09635	*NS*	*NS*	*NS*	*NS*	*89*	*69.55*	*NS*	*NS*	*79*	*68.27*	*NS*	*NS*	*79*	*67.89*
N. moolapensis NMLP_RS00490	-	-	*96*	*75.50*	*15*	*85*	*88*	*66.46*	*NS*	*NS*	*85*	*66.08*	*NS*	*NS*
Hbr. ezzemoulense EO776_RS05910	-	-	-	-	*NS*	*NS*	*82*	*67*	*NS*	*NS*	*81*	*65.75*	*NS*	*NS*
Hbr. ezzemoulense EO776_RS13550	-	-	-	-	-	-	*NS*	*NS*	*93*	*70.17*	*NS*	*NS*	*86*	*70.19*

Hrc. hispanica HISP_RS09995	-	-	-	-	-	-	-	-	*NS*	*NS*	*100*	*93.37*	*NS*	*NS*
Hrc. hispanica HISP_RS12510	-	-	-	-	-	-	-	-	-	-	*NS*	*NS*	*100*	*95.27*
Hrc. japonica C444_RS06270	-	-	-	-	-	-	-	-	-	-	-	-	*NS*	*NS*
